# Exposure-based cognitive behavioral therapy delivered by assertive community treatment teams for severe mental illness with symptoms of anxiety: a cluster randomized controlled trial

**DOI:** 10.1017/S0033291726103365

**Published:** 2026-03-13

**Authors:** Sayaka Sato, Asami Matsunaga, Makoto Ogawa, Masashi Mizuno, Akiko Kikuchi, Hiroaki Kumano, Sosei Yamaguchi, Chiyo Fujii

**Affiliations:** 1 Department of Community Mental Health and Law, National Institute of Mental Health, NCNP, Japan; 2 University of Tsukuba, Japan; 3 Musashino University, Japan; 4 Waseda University, Japan

**Keywords:** severe mental illness, schizophrenia, anxiety, exposure-based cognitive behavioral therapy, assertive community treatment, randomized controlled trial, community mental health, psychosocial intervention, implementation, personal recovery, quality of life, cost-effectiveness

## Abstract

**Background:**

Individuals with severe mental illnesses (SMIs) experience anxiety that impairs functioning and quality of life. This cluster randomized trial evaluated exposure-based cognitive behavioral therapy (ebCBT) integrated into assertive community treatment (ACT) teams to reduce anxiety.

**Methods:**

Fifteen ACT teams were allocated to ebCBT + ACT (k = 8, n = 50) or ACT-only (k = 7, n = 43). The intervention followed four steps: situation identification, four-component analysis (behavior, cognition, emotion, physical symptoms), psychoeducation, and graded exposure. Staff received 50 h training and bimonthly supervision over 12 months. Co-primary outcomes were trait and social anxiety; secondary outcomes were psychiatric symptoms, functioning, quality of life, and recovery.

**Results:**

The ebCBT + ACT group showed significant improvements in State–Trait Anxiety Inventory–Trait scores at 12 months (AMD = −5.30, 95% CI = −8.71 to −1.90, p = 0.002, d = −0.64) and 18 months (AMD = −7.22, 95% CI = −12.1 to −2.34, p = 0.004, d = −0.60). Brief Fear of Negative Evaluation scores showed near-significant improvement at 18 months (AMD = −3.70, 95% CI = −7.44 to 0.04, p = 0.052, d = −0.40). Secondary outcomes, including global functioning, recovery, and quality of life, also improved. Cost-effectiveness analyses indicated favorable cost-effectiveness for anxiety outcomes.

**Conclusions:**

Embedding ebCBT within ACT services may reduce anxiety-related fear and avoidance and enhance recovery-related outcomes in individuals with SMI. These findings support the feasibility and clinical value of integrating structured psychological interventions into intensive community-based outreach services.

## Introduction

A systematic review reported that ~40% of individuals diagnosed with schizophrenia spectrum disorders experience clinically significant anxiety, including comorbid anxiety disorders (Braga, Reynolds, & Siris, 2013). Anxiety-related concerns in individuals with severe mental illness (SMI) are often embedded in social and environmental contexts, including self-stigma and perceived social threat (Fond et al., 2023). Anxiety-related avoidance may be further shaped by broader cognitive patterns, such as defeatist beliefs, which have been shown to correlate with anxiety-related distress (Grant et al., 2009). Because these anxiety experiences are closely linked to other severe psychiatric symptoms, reduced quality of life, and adverse outcomes, community mental healthcare providers, including care managers in assertive community treatment (ACT), have sought effective therapeutic interventions. Cognitive behavioral therapy (CBT) is among the most effective interventions for treating SMIs (Solmi et al., 2023).

ACT is a multidisciplinary, team-based approach for severe psychiatric conditions that originated in the United States (Dixon, 2000) and provides comprehensive, individualized care outside formal clinical settings. In Japan, ACT programs, implemented with reference to the Dartmouth fidelity scale (Teague, Bond, & Drake, 1998), serve individuals with SMI characterized by high service needs and substantial functional impairment (Ito, 2008; Yamaguchi et al., 2020). However, CBT has traditionally been delivered in clinics or agency-based settings, and its provision within community outreach services remains limited (Thornicroft & Susser, 2001). This suggests that people with SMI face barriers to accessing effective interventions for psychological distress and maladaptive behaviors, which may be addressed through community-based CBT.

Several studies have examined the effects of CBT on SMI and its modes of delivery. Systematic reviews demonstrate small effects of CBT on psychiatric symptoms, functioning, and distress (Bighelli et al., 2018; Jones et al., 2018; Laws et al., 2018), as well as medium to large effect sizes for reducing general and social anxiety (Jones et al., 2018; Michail, Birchwood, & Tait, 2017). In prior research, CBT has often been used as an umbrella term encompassing heterogeneous intervention components, including CBT for psychosis, skills training, and illness self-management. However, the effects of these CBT approaches on anxiety and fear in individuals with SMI may be limited, suggesting that exposure-based CBT (ebCBT), an approach inherently effective for these symptoms, may be particularly relevant. Despite these potential benefits, service disengagement remains common. Although meta-analytic evidence indicates that individuals with severe psychiatric symptoms show high treatment response (Bighelli et al., 2018), they also demonstrate low treatment engagement (Dixon, Holoshitz, & Nossel, 2016). Engagement is associated with staff time and commitment, communication beyond medication-focused interactions, and the quality of therapeutic relationships (Singh, 2000; Thornicroft, Szmukler, Mueser, & Drake, 2011). In this context, ACT, a team-based service model characterized by intensive outreach and engagement, represents a promising approach for engaging individuals with SMI(Gowda & Isaac, 2022). Building on outreach-oriented perspectives in CBT delivery (Beck & Naz, 2019), integrating CBT within ACT services represents a promising strategy for improving access to psychological interventions for this population. Anxiety in SMI can be conceptualized at multiple, interrelated levels.

Anxiety may arise in social and environmental contexts, including stigma-related fears, negative self-evaluative cognitions driven by social comparison, and restricted life opportunities such as education, employment, and social participation. Although these experiences do not necessarily meet criteria for formal anxiety disorders, repeated exposure in daily life may contribute to learned fear responses and avoidance behaviors that interfere with functioning. Over time, these behavioral patterns may also be reflected in relatively stable anxiety-related traits. Prior studies have examined CBT training for outreach staff and reported improvements in staff confidence and aspects of service user outcomes, including insight, negative symptoms, and relapse-related indicators (Malik et al., 2009; Pinninti, Fisher, Thompson, & Steer, 2010; Turkington et al., 2006).

Despite these findings, evidence for CBT within outreach service settings, including ACT, remains insufficient. CBT is highly effective for anxiety-related disorders (van Dis et al., 2020) and promising for anxiety in individuals with SMIs (Michail, Birchwood, & Tait, 2017). When targeting anxiety-related symptoms, behavioral therapy techniques based on exposure methods may be effective (Carpenter et al., 2018; Szuhany & Simon, 2022). However, previous studies incorporating CBT into outreach services did not employ ebCBT or evaluate anxiety-related outcomes. Moreover, although ACT serves individuals with SMIs likely to experience disengagement, no randomized controlled trials (RCTs) have examined the effectiveness of CBT integration into ACT. In addition, prior studies indicate that CBT delivery in outreach or ACT settings generally requires staff training and supervision (Malik et al., 2009; Pinninti, Fisher, Thompson, & Steer, 2010; Turkington et al., 2006). A cluster RCT (cRCT) design is well-suited for evaluating educational or training interventions by preventing contamination across groups. However, no study has used a cRCT design to investigate the effectiveness of combined CBT and outreach service interventions, including ACT. To address these gaps, we conducted a project providing CBT training and ongoing supervision to ACT team staff. This study aimed to evaluate the effectiveness and cost of CBT delivered by ACT staff to service users using a cRCT design.

## Methods

### Study design and setting

We conducted a cRCT with a parallel-group design, site-level 1:1 allocation, and 18-month follow-up in Japan, adhering to consolidated cRCT reporting standards (Campbell, Piaggio, Elbourne, & Altman, 2012). We recruited 20 ACT teams from April 1, 2016. Eligibility required registration with the Community Mental Health Outreach Association, which provides outreach services and conducts ACT fidelity reviews. Fifteen teams participated. CBT training was provided to staff in the intervention arm, who delivered CBT for 1 year. Outcomes were assessed at baseline (T1), 12 months (T2), and 18 months (T3). The study was conducted from September 1, 2016, to March 31, 2019, with ethics approval from the National Institute of Mental Health, National Center of Neurology and Psychiatry, Japan (UMIN000026355).

### Participants

Service users in ACT teams were recruited between September 1 and 30, 2016. Inclusion criteria were: (1) age ≥ 20 years, (2) staff-assessed anxiety interfering with daily life, and (3) written informed consent. Participants were excluded if they had a primary diagnosis of an organic or personality disorder. For criterion (2), ACT staff assessed whether service users exhibited: (A) difficulty leaving the house due to anxiety, (B) difficulty ceasing compulsive behaviors (e.g. checking, hoarding, and washing), or (C) difficulty initiating actions due to anxiety-related concerns. These anxiety-related difficulties were selected based on findings from a preliminary survey of Japanese ACT teams conducted as part of a government-funded research project (Sato, 2016). Participant identification relied on clinical judgment by ACT staff, focusing on observable anxiety-related fear or avoidance that interfered with daily functioning in community settings, rather than on formal diagnostic criteria for anxiety disorders or standardized screening thresholds. Anxiety experiences were considered clinically relevant regardless of diagnostic status and informed both participant selection and identification of intervention targets. No standardized anxiety scales were used for screening; instead, case managers identified eligible participants through routine observations during daily support, selecting individuals who explicitly expressed anxiety-related difficulties (e.g. fear of going outside and repetitive gas checking due to fire concerns). Written informed consent was obtained from all participants.

### Intervention and procedure

The ebCBT targeted anxiety-related responses and avoidance patterns maintained by cognitive distortions and prior learning experiences; anxiety arising from psychiatric symptoms or cognitive dysfunction was not the primary target. Initial steps included identifying anxiety-generating situations, documenting four elements (behavioral responses, related cognitions, emotional reactions, and physical symptoms, such as avoidance, anxious thoughts, fear, and somatic sensations), and explaining that anxiety involves changes beyond emotions alone. Psychoeducation on anxiety was then provided based on personal cognitions in anxious situations. Case formulation examined relationships among the four elements, explaining their mutual influence and how behavioral modification could positively affect cognition, emotions, and physical symptoms, thereby enhancing motivation for exposure. Finally, anxiety-inducing situations were listed to construct hierarchies based on Subjective Units of Disturbance, and exposure was initiated from lower-ranked situations in small steps. When participants exhibited prominent negative symptoms, such as reduced motivation or behavioral inactivity, these were addressed by adjusting intervention delivery (e.g. pacing, step size, and timing of exposure tasks) rather than altering intervention content.

The intervention was implemented in two stages. First, a clinical psychologist trained the staff, after which the trained staff delivered ebCBT to service users. Within each team, staff who expressed interest in delivering CBT and participating in training were recruited and designated as CBT providers, with no specific professional requirements imposed. To support treatment delivery and ensure intervention quality, an ongoing consultation system via email or telephone and a 1-year training and supervision program were implemented. Supervision consisted of face-to-face sessions every 2 months, and in some cases, a case manager other than the designated CBT provider participated on an ad hoc basis. During supervision, participant case reports were presented and discussed for ~30–40 min. Training and supervision were conducted by a clinical psychologist (PhD, >10 years of research and clinical experience). Cases were selected based on clinical need, including guidance during the ebCBT process, and discussed collaboratively by the ebCBT provider and case managers within each team. Depending on the team, supervision focused on the same case across sessions or different cases as clinically indicated. The psychologist and two supervisors provided ~50 h of comments and consultations. CBT was offered exclusively to study participants, and ebCBT was implemented by single staff members under regular supervision. In some cases, other ACT staff members (e.g. case managers), under the guidance of the primary ebCBT provider, delivered limited initial components, such as listing behaviors and cognitions and providing basic anxiety psychoeducation, and collaborated on homework through routine services. Planned exposure tasks were primarily conducted by participants in their daily lives. In some cases, when clinically appropriate, ACT staff supported or accompanied participants during routine visits; however, this support was intended solely to complement exposure tasks planned by the ebCBT provider. To maintain quality, trained staff delivered ebCBT using a four-step process. One author confirmed treatment fidelity (SS). At the T2 and T3 assessment time points, staff provided written reports regarding implemented program steps. Bimonthly case reviews included supervision by two experienced therapists: a psychosomatic physician and clinical psychologist (KH), former president of the Japanese Association of Cognitive and Behavioral Therapy and certified CBT therapist, and a clinical psychologist (AK) with a PhD, expertise in CBT for psychosis, and more than 15 years of supervision experience.

### Outcomes

In this study, the primary outcomes were trait anxiety, assessed using the State–Trait Anxiety Inventory–Trait Form (STAI-T) (Spielberger, Gorsuch, & Lushene, 1970), and fear of negative evaluation, measured by the Brief Fear of Negative Evaluation Scale (BFNE) (Leary, 1983), selected to capture relatively stable anxiety-related tendencies and social-evaluative anxiety associated with fear and avoidance in daily community life. Secondary outcomes included psychiatric symptoms (Brief Psychiatric Rating Scale [BPRS]) (Overall & Gorham, 1962), global functioning (Global Assessment of Functioning [GAF]) (American Psychiatric Association, 1994), quality of life (World Health Organization Quality of Life-26 [WHO-QOL26]) (World Health Organization, 1998), and personal recovery (Recovery Assessment Scale [RAS]) (Corrigan et al., 2004). All Japanese versions demonstrated adequate reliability and validity for clinical research use (Chiba, Miyamoto, & Kawakami, 2010; Nakazato & Mizuguchi, 1982; Sasagawa et al., 2004; Yokoyama & Origasa, 2003). Higher scores on STAI-T and BFNE indicated greater severity, while higher scores on RAS indicated greater recovery. WHO-QOL26 assessed four domains: physical, psychological, social relationships, and environmental. Clinicians rated BPRS, and ACT staff rated GAF, unblinded to treatment assignments, while WHO-QOL26, RAS, STAI-T, and BFNE were patient-reported measures. ACT teams managed scale administration. BPRS was rated during routine medical examinations. Case managers provided support as needed during questionnaire completion. Readmissions were recorded over an 18-month follow-up period.

Service costs were calculated from healthcare and social service perspectives. ACT service provision was recorded using the Client Service Receipt Inventory Japanese version (CSRI-J) (Yamaguchi et al., 2012). The CSRI-J included social benefits, community services, hospitalization, and medical care costs. Medication costs were derived from receipt data.

CBT fidelity was assessed using the Cognitive Therapy Rating Scale (CTRS) (Young & Beck, 1980). To avoid overwhelming ACT participants, staff considered approaching participants for audio recordings ~6 months after ebCBT initiation. This time point was selected to avoid disrupting the early phase of ACT service delivery, as ACT teams typically require time to establish therapeutic relationships and trust with service users. Service delivery conversations were audio-recorded and independently evaluated by two authors: a physician (MO) and a psychologist (MM). ACT contacts were prospectively recorded using monthly contact logs, in which staff documented the primary purpose of each visit. Contacts were summarized by month and classified according to whether their primary focus corresponded to early ebCBT components (problem structuring of anxiety-provoking situations and psychoeducation), advanced ebCBT components (case formulation and exposure), or other routine ACT support.

### Sample size and sampling methods

Few studies examined CBT effects on trait anxiety in community samples of individuals with SMI, so the sample size was based on co-primary FNE outcomes. Previous research reported a 1.05 effect size between the CBT and control groups (Kingsep, Nathan, & Castle, 2003). Detecting this difference with 90% power and 5% significance required 25 participants per group. cRCT calculations multiply sample size by the design effect (1 + [*m* − 1]*ρ*), where *m* is the average cluster size, and *ρ* is the intracluster correlation coefficient (ICC) (Malik et al., 2009). Assuming an ICC of 0.1 and seven clusters per arm, 40 participants per arm are required. With 25% attrition, the final sample required was 50 per arm (100 total).

### Randomization and masking

ACT team-level randomization was stratified by staff numbers and the previous year’s ACT fidelity scores, using mean values as cutoffs. An independent researcher received anonymized team data and generated random allocation sequences using Stata (StataCorp LLC, College Station, TX, USA). Teams were assigned to ACT-only (control) or ebCBT + ACT (intervention) groups. An independent statistician conducted blinded analyses.

### Statistical analyses

To examine intervention effects, random intercept and random slope models were used to analyze continuous variables. All models used restricted maximum likelihood estimation and Satterthwaite degree-of-freedom corrections, which are recommended for studies with a small number of clusters (Leyrat, Morgan, Leurent, & Kahan, 2018). To account for individual differences among participants and team factors, these variables were included as random effects. Adjusted mean differences (AMDs) and effect sizes were then computed for each outcome scale, adjusting for baseline scores. For rehospitalization during the study period, mixed-model logistic regression was used. Intention-to-treat analysis was applied throughout. Sensitivity analyses adjusted for age, sex, diagnosis of mood disorders, marital status, education level, and living situation. An additional analysis examined whether participants with higher baseline anxiety in the ebCBT + ACT group showed greater improvement in STAI-T and BFNE scores. Low and high anxiety groups were defined using cutoff points (high group STAI-T, Male ≥ 49, Female ≥ 48, BFNE ≥ 39) as the independent variable, with score changes from baseline to 18 months as the dependent variable. Generalized linear models were additionally computed, adjusting for the same covariates. For service costs, the incremental cost-effectiveness ratio (ICER) was calculated for the mean improvement in STAI-T scores (a one-point change indicated improvement). The ICER was defined as the difference in mean costs between groups divided by the difference in group effects. As cost data were available only for trial completers, ICER analyses included participants who completed the STAI and CSRI at the 18-month follow-up. All analyses were performed using Stata version 15. Statistical significance was set at 5% (*p* < 0.05).

## Results

### Recruitment

**Figure 1.**
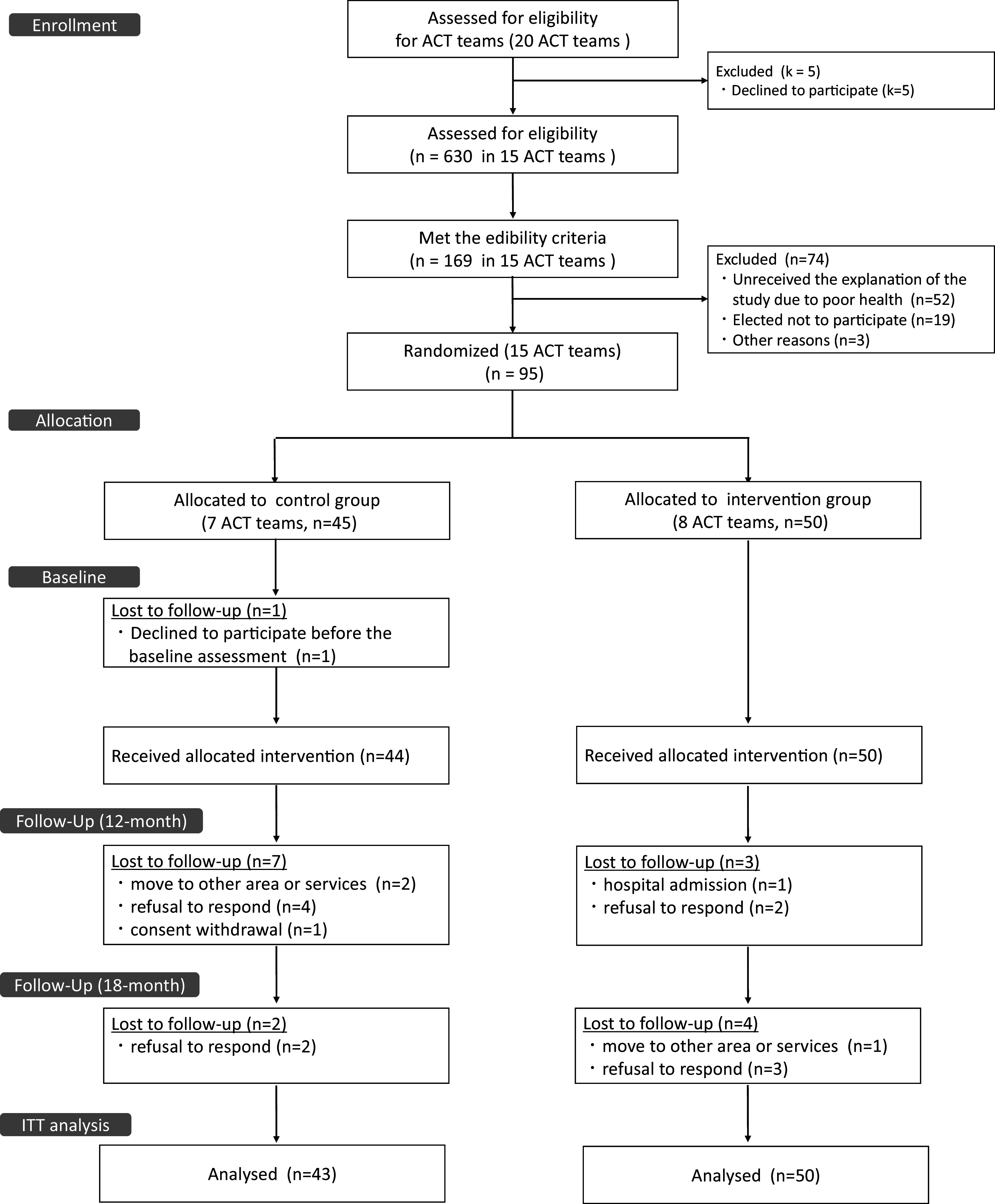
CONSORT flow diagram showing participant flow through recruitment, cluster allocation, follow-up, and analysis in the cluster randomized controlled trial.

Figure 1 illustrates the participant recruitment flow. A total of 630 individuals receiving ACT across 15 teams were assessed for eligibility. Of these, 169 patients were identified with anxiety-related symptoms, and 95 consented to participate. The 15 ACT teams were randomly assigned to the ebCBT + ACT group (*n* = 8) or the ACT-only group (*n* = 7) (Online Supplementary Table 1). In the ACT-only group, one participant declined the survey before receiving the intervention, and no data were obtained; 50 and 44 participants received the intervention in the ebCBT + ACT and ACT-only groups, respectively. During the 18-month follow-up, seven participants in the ebCBT + ACT group and nine in the ACT-only group were lost to follow-up. Of these, one participant in the ACT-only group withdrew consent. Consequently, 93 participants were included in the Intention to treat analysis (50 in the ebCBT + ACT group and 43 in the ACT-only group).

### Sample characteristics and outcome assessment at baseline

The mean age of participants was in the 40s (Table 1). Approximately half of the participants in the ebCBT + ACT group and ~60% in the ACT-only group were male. Schizophrenia was the most frequent diagnosis in both groups, although the ebCBT + ACT group also included participants with affective disorders. Correspondingly, differences in antidepressant dosage were observed between groups. Among the three anxiety types that were problematic in daily life and served as inclusion criteria, the most frequent was difficulty taking action due to anxiety-related thoughts or concerns. At baseline, STAI-T (*t* = −3.517, *p* = 0.001) and BFNE (*t* = −2.136, *p* = 0.035) scores were significantly higher in the ebCBT + ACT group than those in the ACT-only group. The ebCBT + ACT group also had lower scores on the RAS (*t* = 2.873, *p* = 0.005) and the WHO-QOL26 physical (*t* = 2.146, *p* = 0.035) and psychological domains (*t* = 2.946, *p* = 0.004).

**Table 1.** Sample characteristics of the participants

	ACT-only		ebCBT ± ACT		Statistical test	*p*
	(*n* = 44)		(*n* = 50)			
Age, mean (sd)	45.11	(9.93)	42.16	(11.56)	*t* = 1.319	0.19
Sex, *n* (%)						
Male	27	(61.40)	24	(48.00)	χ2 = 1.684	0.214
Diagnosis, *n* (%)					χ2 = 4.971	0.290
Schizophrenia	40	(90.90)	40	(80.00)		
Affective disorders	0	0.00	5	(10.00)		
Bipolar disorders	2	(4.50)	2	(4.00)		
Anxiety disorders	1	(2.30)	2	(4.00)		
Developmental disorders	1	(2.30)	1	(2.00)		
Illness duration (months), mean (sd)	271.36	(155.20)	215.48	(130.90)	*t* = 1.894	0.061
Duration using ACT services (months), mean (sd)	48.98	(45.41)	31.18	(27.48)	*t* = 2.329	0.022
Admission (times), mean (sd)	5.59	(5.19)	4.62	(6.98)	*t* = 0.757	0.451
Prescription (mg/per day)						
Antipsychotic, mean (sd)	626.36	(398.98)	760.65	(746.00)		
Anxiolytic, mean (sd)	2.61	(5.34)	4.67	(9.84)		
Antidepressant, mean (sd)	1.31	(8.59)	30.61	(74.17)		
Educational type					χ2 = 3.719	0.445
Junior high school	2	(33.33)	4	(66.67)		
High school	19	(41.30)	27	(58.70)		
Vocational school/Junior college	10	(62.50)	6	(37.50)		
University	11	(45.83)	13	(54.17)		
Other	1	(100.00)	0	(0.00)		
Marital status					χ2 = 4.142	0.126
Single	36	(48.65)	38	(51.35)		
Married/living with partner	6	(54.55)	5	(45.45)		
Divorced/widowed	1	(12.50)	7	(87.50)		
Residential status					χ2 = 1.047	0.593
Living alone at home	12	(38.71)	19	(61.29)		
Living with family at home	22	(47.83)	24	(52.17)		
Group/Care home with a supervisor	8	(53.33)	7	(46.67)		
Social participation					χ2 = 4.005	0.135
Volunteer/Peer activities	1	(20.00)	4	(80.00)		
Other	6	(31.58)	13	(68.42)		
None	36	(52.17)	33	(47.83)		

### Effects of adding ebCBT to ACT on each outcome

To examine whether ACT contact intensity differed between groups, an exploratory analysis compared the mean number of staff–participant contacts per month over the first 12 months. No statistically significant difference in mean monthly contact frequency was observed between the ebCBT + ACT group and the ACT-only group (mean ± SD: 9.23 ± 4.76 versus 11.22 ± 6.93; *t* (88) = 1.610, *p* = 0.111). Descriptive summaries of CBT delivery at the team level during the 12-month follow-up are presented in Supplementary Table 4.

For primary outcomes, the ebCBT + ACT group showed significantly greater improvement in STAI-T scores at the 12-month follow-up (AMD = −5.30, 95% confidence interval [CIs] = −8.71, −1.90, *p* = 0.002) and the 18-month follow-up (AMD = −7.22, 95% CI = −12.1, −2.34, *p* = 0.004) compared with the ACT-only group. Effect sizes for STAI-T improvement were moderately large (Cohen’s *d* = 0.64 at 12 months and 0.60 at 18 months). No significant group difference in BFNE scores was observed at the 12-month follow-up, although a near-significant difference was observed at 18 months (AMD = −3.70, 95% CI = −7.44, 0.04, *p* = 0.052, *d* = 0.40) (Table 2). Sensitivity analyses showed similar trends, with a significant difference in BFNE scores at 18 months (AMD = −1.98, 95% CI = −7.45, −0.03, *p* = 0.048). Within the ebCBT + ACT group, participants with higher baseline STAI-T scores showed significantly greater improvement at 18 months than those with lower baseline STAI-T scores (Online Supplementary Table 2), indicating greater benefit among individuals with more severe baseline anxiety. No significant difference in BFNE score change was observed between participants with low and high baseline BFNE scores.

**Table 2.** Co-primary outcomes over 18 months

		ACT-only		ebCBT ± ACT		Adjusted mean difference (95% CI)		*p*	Effect size (95% Cl)	
		Mean	SE	Mean	SE					
STAI-T	Baseline	53.69	0.50	54.05	0.47					
	12-month	53.46	1.29	48.15	1.15	−5.30	(−8.71, −1.90)	0.002	0.64	(0.22, 1.06)
	18-month	54.64	1.86	47.42	1.65	−7.22	(−12.1, −2.34)	0.004	0.60	(0.19, 1.02)
BFNE										
	Baseline	40.84	0.61	41.09	0.58					
	12-month	40.39	1.06	39.09	0.94	−1.30	(−4.08, 1.48)	0.360	0.19	(−0.21, 0.60)
	18-month	41.72	1.42	38.02	1.26	−3.70	(−7.44, 0.04)	0.052	0.40	(−0.01, 0.82)

*Note*: BFNE, Brief Fear of Negative Evaluation Scale; STAI-T, State–Trait Anxiety Inventory-Trait Form.

The secondary outcomes are presented in Table 3. Compared with the ACT-only group, the ebCBT + ACT group showed significant improvements in GAF scores at 12 months (AMD = 6.21, 95% CI = 2.57, 9.83, *p* = 0.001) and 18 months (AMD = 6.27, 95% CI = 1.20, 11.33, *p* = 0.015), as well as in RAS scores at 12 months (AMD = 7.95, 95% CI = 3.34, 12.56, *p* = 0.001) and 18 months (AMD = 7.47, 95% CI = 0.91, 14.02, *p* = 0.026). Improvements in global functioning and personal recovery were sustained across follow-up. For the WHO-QOL26, significant improvements in the ebCBT + ACT group were observed in the social relationships subscale at 18 months (AMD = 0.54, 95% CI = 0.19, 0.90, *p* = 0.003) and the environment subscale at 12 months (AMD = 0.30, 95% CI = 0.09, 0.51, *p* = 0.005) were observed. Improvements in social relationships emerged only at 18 months, suggesting that changes in social functioning may require an extended time to manifest. No significant differences were observed in rehospitalization rates between groups (odds ratio = 0.34, 95% CI = 0.04, 3.45, *p* = 0.362). No significant changes were observed in medication use, including anxiolytics, indicating that improvements were not mediated by pharmacological changes. Sensitivity analyses showed similar trends across outcomes, supporting the robustness of the findings.

**Table 3.** Secondary outcomes over 18 months

		ACT-only		ebCBT ± ACT		Adjusted mean difference (95% CI)		*p*	Effect size (95% Cl)	
		Mean	SE	Mean	SE					
BPRS										
	Baseline	50.14	0.13	50.13	0.12					
	12-month	49.12	1.17	46.37	1.08	−2.75	(−5.88, 0.38)	0.085	0.40	(−0.05, 0.77)
GAF										
	Baseline	37.71	0.68	37.65	0.64					
	12-month	40.71	1.36	46.92	1.25	6.21	(2.57, 9.83)	0.001	−0.69	(−1.11, −0.27)
	18-month	41.94	1.90	48.21	1.75	6.27	(1.20, 11.33)	0.015	−0.50	(−0.92, −0.08)
RAS										
	Baseline	76.04	0.76	75.54	0.70					
	12-month	75.81	1.75	83.76	1.55	7.95	(3.34, 12.56)	0.001	−0.71	(−1.13, −0.29)
	18-month	76.11	2.49	83.57	2.22	7.47	(0.91, 14.02)	0.026	−0.47	(−0.88, −0.05)
WHO-QOL26										
Physical domain										
	Baseline	2.74	0.03	2.71	0.03					
	12-month	2.79	0.06	2.83	0.05	0.04	(−0.12, 0.20)	0.594	−0.11	(−0.52, 0.30)
	18-month	2.70	0.08	2.85	0.07	0.15	(−0.06, 0.37)	0.167	−0.29	(−0.70, 0.12)
Psychological domain										
	Baseline	2.85	0.07	2.65	0.07					
	12-month	2.85	0.08	2.75	0.07	−0.09	(−0.31, 0.12)	0.381	0.18	(−0.22, 0.59)
	18-month	2.82	0.08	2.72	0.07	−0.10	(−0.32, 0.12)	0.360	0.19	(−0.22, 0.60)
Social relationship domain										
	Baseline	2.32	0.06	2.31	0.05					
	12-month	2.29	0.10	2.46	0.09	0.18	(−0.09, 0.44)	0.189	−0.27	(−0.68, 0.14)
	18-month	2.11	0.14	2.65	0.12	0.54	(0.19, 0.90)	0.003	−0.62	(−1.04, −0.20)
Environment domain										
	Baseline	2.92	0.04	2.91	0.03					
	12-month	2.81	0.08	3.11	0.07	0.30	(0.09, 0.51)	0.005	−0.58	(−1.00, –0.16)
	18-month	2.80	0.11	3.09	0.10	0.29	(−0.01, 0.58)	0.056	−0.40	(−0.81, 0.01)

*Note:* BPRS, Brief Psychiatric Rating Scale; GAF, Global Assessment of Functioning; RAS, Recovery Assessment Scale; WHOQOL-BREF, World Health Organization Quality of Life.

### Cost comparison

The cost analysis included 35 and 43 participants in the ACT-only and ebCBT + ACT groups, respectively. The ACT-only group showed a −1.9 change in STAI-T scores with mean costs of $19441.3 (SD = 10082.9), whereas the ebCBT + ACT group showed a 7.3-point improvement and incurred mean costs of $17683.9 (SD = 7747.8) (Online Supplementary Table 3). The mean cost difference between groups was $1757.4 lower in the ebCBT + ACT group. Consequently, the ICER was −191.6, indicating that the ebCBT + ACT group was dominant, being both less costly and more effective than the ACT-only group. This economic advantage appeared to be driven primarily by reduced hospitalization costs and more efficient service utilization in the intervention group.

### Fidelity assessment

Six months after initiation of ebCBT, a fidelity assessment using the CTRS was conducted for two or three participants per team. The mean CTRS score across 17 participants was 36.1 ± 9.5. These scores indicated adequate treatment fidelity, with most sessions meeting accepted standards for CBT delivery. The assessment showed that staff members were able to implement core CBT techniques, including exposure exercises and anxiety hierarchy development, despite limited prior CBT training. However, variability was observed across teams, with scores ranging from 24 to 48, suggesting that additional supervision may benefit some providers in future implementations.

## Discussion

This cRCT examined the clinical and economic impact of ebCBT delivered by ACT teams. The mean number of staff contacts over 1 year was 72.86 ± 50.55. More than 80% of participants completed CBT support, with a dropout rate of 14%. Compared with ACT alone, the ebCBT + ACT group showed significant improvements across several outcomes, including STAI-T, GAF, RAS, and selected WHO-QOL26 domains, particularly at the 18-month follow-up. The addition of ebCBT was especially beneficial for ACT users with high trait anxiety. Moreover, the ICER indicated that ebCBT + ACT achieved lower costs per unit improvement in STAI-T compared with ACT alone.

Implementation of ebCBT alongside ACT resulted in greater reductions in trait anxiety than ACT alone. CBT-based support could be delivered in locations and at times proximal to participants’ anxiety-provoking situations. Exposure techniques are most effective when conducted in environments closely aligned with real-life anxiety triggers. Accordingly, ebCBT effectiveness may have been enhanced by integration within routine ACT service delivery processes. By confronting various scenarios in their homes or communities, often within routine ACT support, participants may have recognized that their anxiety was less impairing in real-life situations. The ebCBT + ACT group demonstrated substantial improvements in social functioning, as assessed by GAF, in addition to reductions in trait anxiety, compared with the ACT-only group. Individuals with SMI often experience cognitive impairment (Regev & Josman, 2020), and talking-based therapies may be particularly challenging for those with higher severity who require ACT services. Exposure-based CBT involves confronting participants with task-related situations, which may facilitate tangible reductions in anxiety. Understanding and implementing CBT processes and techniques can be complex (van Genk et al., 2023). However, ACT staff involved in this study, who were previously unfamiliar with CBT, reported that adopting a simplified ebCBT approach was valuable. This approach may have facilitated effective implementation and contributed to reductions in trait anxiety. The findings also support the cost-effectiveness of the ebCBT + ACT model. Although a more comprehensive economic evaluation is warranted, integrating simplified ebCBT within ACT teams may yield meaningful economic benefits.

Contrary to trait anxiety, no significant between-group differences were observed in social anxiety as measured by BFNE, particularly at the 12-month follow-up. One plausible explanation is that not all participants presented with clinically meaningful social anxiety at baseline, which may have attenuated observable treatment effects. Nevertheless, the ebCBT + ACT group showed a near-significant reduction in BFNE scores at the 18-month follow-up compared with the ACT-only group. Social anxiety typically decreases as individuals increase engagement in social and outdoor activities over time, which may account for the delayed effects observed in this study. Consistent with this interpretation, participants in the ebCBT + ACT group demonstrated improvement in the social relationships domain of WHO-QOL26 at the 18-month follow-up. Within the ACT context, enhancement of social relationships and the associated alleviation of social anxiety may require longer timeframes before meaningful change becomes evident.

Regarding other outcomes, no significant between-group differences were observed in symptom-related measures, including BPRS and the physical and psychological domains of WHO-QOL26. This finding is consistent with prior evidence, as neither ACT nor CBT interventions have consistently demonstrated substantial effects on core psychiatric symptom severity (Bighelli et al., 2018; Dieterich et al., 2017; Jones et al., 2018; Laws et al., 2018). In contrast, the ebCBT + ACT group showed superior outcomes in RAS and the environmental domain of WHO-QOL26 at the 12-month follow-up, which may be partially attributable to reductions in anxiety. One plausible mechanism is that decreased anxiety enabled participants to engage more actively with their surrounding environments, thereby contributing to improvements in quality of life and personal recovery.

Participants may have developed a more positive self-perception and a more favorable outlook toward their surrounding environment as a result of anxiety alleviation achieved through ebCBT. Previous studies have demonstrated associations between personal recovery and anxiety among individuals with SMI (Kraiss et al., 2021; Wood et al., 2017). Accordingly, reducing anxiety through ebCBT may represent a viable strategy to promote individual insight and enhance ACT users’ engagement in community life.

### Strengths and limitations

This study had two primary strengths. First, it focused on anxiety among community-dwelling individuals with SMI who required outreach services at the ACT program level. As this population often disengages from appropriate treatments (Jones et al., 2018), including ebCBT, which is effective in reducing anxiety, these findings highlight the potential of ebCBT for application and delivery within ACT services. Second, this trial employed a cluster design, which helped prevent contamination of interventions between groups.

This study has some limitations. First, baseline differences across several outcome measures were observed between groups. Although these differences were addressed analytically through baseline adjustment and calculation of adjusted means, careful interpretation of the findings remains necessary. Notably, STAI-T and BFNE scores in the ebCBT + ACT group at follow-up were not lower than baseline scores in the ACT-only group. This pattern suggests that ebCBT may not fully alleviate anxiety symptoms among ACT service users in community settings. Second, assessor blinding was not feasible. Practitioners aware of group allocation conducted clinician-rated assessments, including BPRS and GAF. Although most outcome measures were patient-reported, this limitation may have contributed to the overestimation of intervention effects. Third, certain limitations should be noted regarding the fidelity assessment using the CTRS. Participants selected for audio recording were those whom staff judged to be the most approachable when requesting consent, which may have resulted in overestimation of fidelity scores. Because participants were ACT users who require careful attention to preserve therapeutic relationships and because ebCBT was delivered in community settings, it was not feasible to obtain recordings from all participants in a manner comparable to clinic-based trials. Furthermore, fidelity assessment relied on audio-recorded sessions obtained at ~6 months after intervention initiation, rather than across all intervention contacts. As ebCBT was delivered flexibly within routine ACT practice, the content and structure of recorded sessions may have been influenced by participants’ clinical status at the time of recording. When participants experienced symptom exacerbation or acute difficulties, intervention delivery may have appropriately prioritized immediate support over strict adherence to the intervention framework, which could have contributed to variability in fidelity scores. In addition, the intensity and duration of anxiety-focused treatment could not be quantified independently from routine ACT contacts. Although attempts were made to record time devoted specifically to ebCBT sessions separately from usual ACT support, ebCBT was frequently delivered in an integrated and flexible manner during ongoing ACT contacts rather than as clearly delineated stand-alone sessions. Consequently, anxiety-focused interventions were often embedded within routine ACT care in response to participants’ clinical needs, limiting our ability to quantify ebCBT session time independently from usual care. Although the exact duration and intensity of stand-alone ebCBT sessions could not be quantified, ACT contacts were prospectively recorded, and visit frequency was counted and descriptively classified according to primary focus, as presented in the Supplementary Material. In addition, supervision records and clinical discussions provided an overall picture of treatment intensity, allowing characterization of how ebCBT was typically delivered within ACT services. Fourth, the sample size of this study may have been limited. Sample size calculations were based on Kingsep’s study of talking-based group CBT rather than ACT users, which reported relatively large effect sizes. However, significant effects were observed for the primary outcomes, suggesting that sample size limitations may not have substantially influenced the main findings. The cost analysis sample was further restricted to participants who completed follow-up assessments, which may limit generalizability to individuals who discontinued treatment. Fifth, the generalizability of the findings may be limited to ACT staff. Outcomes may differ if the same training is provided to other outreach service staff who do not routinely work with individuals with SMI.

An additional clinical insight from this study is that psychologists’ capacity to provide supervision or consultation to other professional groups is inherently limited, which may influence ebCBT effectiveness. Furthermore, the absence of factors that enhance team motivation, including financial incentives, may pose barriers to ebCBT implementation. As a potential strategy to address these challenges, psychologists engaged in community-based services could oversee multiple teams and provide structured supervision across teams, thereby supporting broader implementation.

## Conclusion

This cRCT evaluated the clinical efficacy and cost-effectiveness of ebCBT as implemented by trained ACT team members. The findings suggest that integrating ebCBT into ACT represents a promising approach for reducing trait anxiety among ACT service users without additional costs. Modification of social anxiety and improvements in perceived social relationships may also be achievable through integration of ebCBT with ACT; however, such changes may require time following intervention completion to become evident. Furthermore, anxiety reduction through ebCBT may be associated with improved functioning and enhanced personal recovery as perceived by individuals. Overall, simplified CBT strategies, particularly those incorporating exposure techniques, appear effective in supporting individuals with SMI within community mental health service settings.

## Supporting information

10.1017/S0033291726103365.sm001 Sato et al. supplementary material Sato et al. supplementary material

## Data Availability

The datasets generated and/or analyzed during this study are not publicly available due to the applicable Ethical Guidelines for Medical and Health Research Involving Human Subjects and the conditions of ethical committee approval. However, the data are available from the corresponding author upon reasonable request.

## Supplementary material

The supplementary material for this article can be found at http://doi.org/10.1017/S0033291726103365.
