# Supplementary material for: Exposure-based cognitive behavioral therapy delivered by assertive community treatment teams for severe mental illness with symptoms of anxiety: a cluster randomized controlled trial
Source: Psychol Med. 2026 Mar 13;56:e70. doi: 10.1017/S0033291726103365 (PMC13079238; doi:10.1017/S0033291726103365)
Supplement: Sato et al. supplementary material [file S0033291726103365sup001.docx]

**Online Supplementary Tables 1. Characteristics of the participating assertive community treatment teams**

|  | **ACT only (n=7)** | | **ebCBT+ACT (n=8)** | |
| --- | --- | --- | --- | --- |
|  | **n/mean** | **%/sd** | **n/mean** | **%/sd** |
| Number of cases, mean (sd) | 41.00 | (15.45) | 45.13 | (16.81) |
| ACT fidelity score, mean (sd) | 3.89 | (0.18) | 3.84 | (0.19) |
| Type of institution, n (%) |  |  |  |  |
| Hospital | 1 | (14.29) | 2 | (25.00) |
| Clinic | 4 | (57.14) | 5 | (62.50) |
| Others | 2 | (28.57) | 1 | (12.50) |

**Online Supplementary Tables 2: 18month outcomes by baseline anxiety level in the ebCBT + ACT group**

|  | **n** | **Mean** | **SD** | **Coefficient** | **(95%CIs)** | **P** |
| --- | --- | --- | --- | --- | --- | --- |
| **STAI-T** |  |  |  |  |  |  |
| Low score group (Male <=48, Female <=47) | 9 | 1.1 | 4.7 | -13.0 | (-20.9, -5.0) | 0.001 |
| High score group (Male >=49, Female >=48) | 35 | -9.3 | 10.5 |  |  |  |
| **BFNE** |  |  |  |  |  |  |
| Low score group (<=38) | 9 | -0.8 | 6.3 | -5.5 | (-12.9, 1.8) | 0.139 |
| High score group (>=39) | 35 | -3.7 | 8.9 |  |  |  |

BFNE, Brief Fear of Negative Evaluation Scale; STAI-T, State-Trait Anxiety Inventory-Trait Form

Note. Coefficients represent adjusted mean differences in 18-month outcomes between participants with high and low baseline anxiety levels, defined using mean splits of each anxiety measure.

**Online Supplementary Tables 3. Team-level descriptive summary of CBT delivery during the 12-month follow-up period**

| team | Number of  participants | CBT-focused contacts  (first phase) | CBT-focused contacts  (second phase) | Total contacts |
| --- | --- | --- | --- | --- |
| 1 | 4 | 0.92 | 0.88 | 10.02 |
| 2 | 6 | 3.38 | 1.96 | 7.78 |
| 3 | 10 | 2.28 | 0.98 | 5.93 |
| 4 | 6 | 0.15 | 0.57 | 3.18 |
| 5 | 6 | 0.19 | 0.47 | 8.58 |
| 6 | 7 | 1.08 | 1.00 | 15.78 |
| 7 | 4 | 0.48 | 0.96 | 10.92 |
| 8 | 6 | 0.86 | 0.49 | 8.89 |

Note. Values represent mean monthly frequencies summarized over the 12-month follow-up period. CBT-focused contacts were counted only when the primary purpose of the visit was CBT; brief CBT-related discussions embedded within routine ACT support were not included.

**Online Supplementary Tables 4.** **Calculation of incremental cost effectiveness ratio (ICER)**

|  | **ACT only** | | **ebCBT+ACT** | |
| --- | --- | --- | --- | --- |
|  | **mean** | **SD** | **mean** | **SD** |
|  | **n=35** | | **n=43** | |
| **STAI-T** |  |  |  |  |
| Baseline | 48.9 | 11.2 | 57.8 | 11.2 |
| 18-month | 50.8 | 10.7 | 50.5 | 10.2 |
| Mean difference within the group  (Baseline - 18-month) | -1.9 |  | 7.3 |  |
| **Total cost (US $)** | 19441.3 | 10082.9 | 17683.9 | 7747.8 |

STAI-T: State-Trait Anxiety Inventory-Trait Form
